# Vitamin D and Neurodegenerative Diseases Such as Multiple Sclerosis (MS), Parkinson’s Disease (PD), Alzheimer’s Disease (AD), and Amyotrophic Lateral Sclerosis (ALS): A Review of Current Literature

**DOI:** 10.1007/s13668-025-00663-y

**Published:** 2025-06-04

**Authors:** Zehra Savran, Saltuk Bugra Baltaci, Tugce Aladag, Rasim Mogulkoc, Abdulkerim Kasim Baltaci

**Affiliations:** 1 Medical Faculty, Department of Physiology, Selçuk University, Konya, 42250 Turkey; 2 Medical Faculty, Department of Physiology, Istanbul Medipol University, Istanbul, Turkey

**Keywords:** Neurodegenerative diseases, Multiple sclerosis, Parkinson’s disease, Alzheimer’s disease, Amyotrophic lateral sclerosis, Vitamin D

## Abstract

**Purpose of Review:**

This review explores the role of Vitamin D3 and its derivatives as inhibitors of pathological metabolic modifications in neurodegenerative diseases. The manuscript investigates how Vitamin D3 impacts neuronal calcium regulation, antioxidative pathways, immunomodulation, and neuroprotection during detoxification, beyond its known functions in intestinal, bone, and kidney calcium and phosphorus absorption, as well as bone mineralization.

**Recent Findings:**

Recent studies have highlighted the synthesis of the active metabolite 1,25(OH)2D3 (vitamin D) in glial cells via the hydroxylation process of CY-P24A1, an enzyme in the cytochrome P450 system in the brain. The effects of vitamin D occur through the vitamin D receptor (VDR), a nuclear steroid receptor, which has been identified in various brain regions, including the cerebellum, thalamus, hypothalamus, basal ganglia, hippocampus, olfactory system, temporal, and orbital regions. Neurodegeneration is primarily associated with oxidative stress, protein aggregation, neuroinflammation, mitochondrial dysfunction, apoptosis, and autophagy changes, all of which Vitamin D and VDR are believed to influence.

**Summary:**

Vitamin D and VDR are recognized as both environmental and genetic factors in the etiopathogenesis of neurodegenerative diseases such as Multiple Sclerosis (MS), Parkinson’s Disease (PD), Alzheimer’s Disease (AD), and Amyotrophic Lateral Sclerosis (ALS). A deficiency in Vitamin D is postulated to have detrimental effects on the brain and other diseases throughout various stages of life. This review consolidates findings from clinical and experimental studies, as well as past publications, focusing on the implications of Vitamin D deficiency in these neurodegenerative conditions. Current articles published in PubMed were extensively considered for this review.

**Graphical Abstract:**

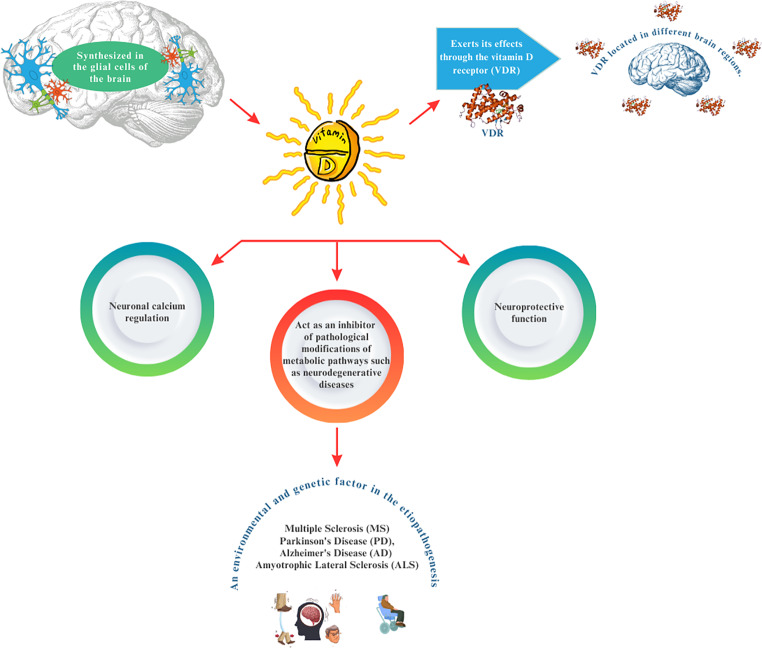

## Introduction

The discovery of vitamin D’s systemic effects has led to new searches/studies on the role of vitamin D in modulating physiological and pathological processes, which has led to the need for further studies in the prevention and treatment of these diseases.

## Vitamin D

Vitamin D is found in two forms in the body: vitamin D2 and vitamin D3. Vitamin D3 is derived from the action of ultraviolet rays from the sun by acting on a cholesterol derivative in the skin. Around 90% of the vitamin D requirement in the human body is achieved by endogenous production dependent on sun exposure [1]. Vitamin D3 is also available from natural sources such as codfish, fish oil, eggs, and fortified milk. Vitamin D2 is only widely available from vegetable diets. Endogenous vitamin D synthesis is the minimum erythema dose (MED) [2]. At the beginning of metabolic activation of Vitamin D undergoes the hydroxylation of 25 carbons, mainly in the liver. Regarding the metabolism in the brain, it has been shown that the cytochrome P450 enzyme systems CYRL-P24A1 synthesize active metabolite D3 by the hydroxylation process in glial cells [3]. D3 shows the effect of the vitamin D receptor (VDR), this receptor has formed a nuclear steroid receptor [4]. Vitamin D and its metabolites reach target tissues and bind with VDR, a transcription factor of the nuclear receptor family in the cytosol. VDR was also detected in the different brain regions of the central nervous system, such as the cerebellum, thalamus, hypothalamus, posterior ganglion, hippocampus, olfactory system, and temporal and orbital regions of the brain [5]. It has been emphasized that vitamin D and VDR levels may be environmental and genetically influential factors in the etiopathogenesis of various neurodegenerative diseases such as MS, PD, AD, and ALS [6]. 1.25 (OH) 2D3 has been shown to have a strong regulatory effect in signal transduction of neuron growth factor (NGF), especially prominent in neurons, and thereafter, it may be important in the development of neurons in the brain.

## Vitamin D and Neurodegenerative Diseases

Research has shown a connection between vitamin D deficiency and several neurological conditions, including Parkinson’s disease, Alzheimer’s, and multiple sclerosis. Its neuroprotective effects are attributed to its immunomodulatory and anti-inflammatory properties. Vitamin D may alleviate neuroinflammation and oxidative stress, support neurotrophic factor production, regulate neurotransmitter synthesis, and protect the blood-brain barrier, leading to improved clinical outcomes and extended disease progression [7].

Sufficient levels of vitamin D may contribute to the prevention and management of depression and cognitive dysfunction in older adults [8].

## Vitamin D and Parkinson’s Disease (PD)

PD is a common, progressive neurodegenerative disorder that leads to disability in older people [9]. In this condition, nerve cells in the brain cannot produce enough dopamine. Among the most common symptoms of PD are tremors, stiffness in arms, legs, and trunk, weakened balance and coordination, and slow movements. There may also be problems such as depression, sleep, and dysphagia [10]. Parkinson’s disease is a neurodegenerative disease characterized by dopaminergic cell loss or deficiency in the pars compacta part of the substantia nigra [11]. The brain of these patients also shows neuroinflammation, oxidative stress, and mitochondrial dysfunction. In these patients, vitamin D treatment protects astrocytes against free oxygen radicals and improves mitochondrial function by reducing oxidative stress and NF-kB and Nrf2 expression [11].

Insufficient vitamin D may lead to a significant risk for chronic neurodegeneration. Although it is unclear how vitamin D protects from PD, one of the possible explanations is likely the neuroprotective effects that may be caused by the antioxidative system, calcium regulation, immunomodulation, increased neurotransmission, and detoxification [12]. It is predicted that chronic vitamin D deficiency causes dopaminergic neuron loss in the substantia nigra region and may cause Parkinson’s disease in the later stages [13]. Evatt et al. [14] found that the prevalence of vitamin D deficiency in the Parkinson’s cohort was significantly higher [14]. In the study of Sanchez et al. [15], it was reported that GDNF expression increased with 1.25 (OH) 2D3 before application and rats with Parkinson’s model reduced the decrease in thyroxine hydroxylase (TH) immunoreactivity in substantia nigra [15]. This suggests that 1.25 (OH) 2D3 may help prevent dopaminergic neuronal damage. In a study of the Chinese population, VDR gene polymorphism and Parkinson’s disease were not correlated [16]. The nigrostriatal pathway is the primary target of neurodegeneration in Parkinson’s because there are many vitamin D receptors here [17]. Vitamin D is also important for the problem of excessive L-DOPA use and, therefore, for PH management [17]. Vitamin D is associated with brain development, maturation, and neurodegenerative diseases such as PD [18]. The neuroprotective effect of vitamin D is achieved by enhancing the effect of neuroprotective factors, nerve growth factors, or protection against cytotoxicity. Abnormal D activation causes failure of the dopaminergic system through Klotho deficiency [19].

It has been determined that the presence of mutational variants and VDR nucleotide polymorphism is associated with neurological diseases [20]. It also revealed that there is a relationship between low 25-hydroxyvitamin D concentration and the onset and development of PD [21]. Cumulative neurodegeneration in PD is accompanied by neuroinflammation and endothelial vascular disruption [22]. The presence of VDRs here also reveals their role in endothelial function. At the same time, increased cytokines play a role in the dyskinesia that occurs in PD, and these are molecules such as TNF-α and IL-6 [23]. Kountouras et al. [24] pointed out that non-beneficial bacteria such as Helicobacter pylori trigger neurodegeneration and that vitamin D supplementation may have a positive role in this case. It has been emphasized that D3 treatment reduces dopamine loss in the substantia nigra and that preventing microglia activation is important in this [25]. The positive effect of vitamin D on L-type voltage-sensitive calcium channels, nerve growth factor, and matrix metalloproteins in Parkinson’s disease was determined [26]. Among the different isoforms of vitamin D in PD, D3 has been emphasized as the most important candidate because it has appropriate receptors in the central nervous system.

Vitamin D3 shows anti-inflammatory and antioxidant effects via the VD3 receptor. This reveals the potential role of VD3 in the treatment and prevention of neurodegenerative diseases such as PD [27]. Skv et al. [28] reported that low levels of D3 or calcidiol, the major forms of vitamin D in the circulation, are a major risk factor for major neurodegenerative diseases, including PD. Vitamin D Multiple, a neuroactive steroid. It is important in helping to treat and optimize neurodegenerative diseases such as ALS, AD, and PD [29]. Low Klotho protein levels in cerebrospinal fluid are associated with the severity of PD and may be a biomarker in aging-related pathways in PD in the future. It has also been suggested [30]. Vitamin D deficiency in Parkinson’s disease causes a deficiency in dopamine levels and causes synuclein accumulation [31]. In experimental studies, it has been emphasized that Vitamin D application has a neuroprotective effect, and this effect is important because it reduces pro-inflammatory cytokines and upregulates anti-inflammatory cytokines [32]. Astrocytes contribute to α-Synuclein pathology by participating in PD and also provide neuroprotection through α-Synuclein clearance [33]. Vitamin D-activating enzyme CYP27B1 was determined as a subpopulation of astrocytes exclusively in PD. CYP27B1-positive astrocytes could display neuroprotective properties as they sequester α-Synuclein oligomers. Vitamin D3 acts as a regulator of neuroinflammation and immune function. Also, Vitamin D activates cytokine release and regulates nuclear and mitochondrial genes. At the same time, vitamin D impacts neurotransmitter synthesis and brain plasticity. These positions vitamin D as a potential adjunct in treating diseases like Parkinson’s. Recently, the role of vitamin D in intestinal microbiota and serotonin synthesis contributing to psychiatric disorders like schizophrenia and depression has been reported [34].

In an experimental study, the anti-inflammatory effects of vitamin D3 in the PD model were reduced after Treg depletion. These data suggest that the anti-inflammatory and neuroprotective effects of vitamin D3 in PD are associated with its potential to increase Treg expansion [35].

A study has revealed that elevated levels of IL-6 and TNF-α in Parkinson’s disease (PD) patients, along with alterations in visfatin and progranulin levels between PD patients with and without dyskinesia, indicate significant changes in inflammatory biomarkers and adipokines in PD [36].

## Vitamin D and Multiple Sclerosis (MS)

MS is a neurodegenerative disease characterized by impaired immune response to myelin sheaths. Sclerotic plaques are found around the brain neurons due to damage to neurons [37]. The development of treatments that affect peripheral nervous system immunity in the clinical treatment of MS has provided significant progress [38]. Causes demyelination of CNS tissues by triggering a T cell-mediated inflammatory attack with an unknown agent [39]. Regulatory T cell-mediated indirect effects have been predicted in some studies [40]. Regulatory T cells are suppressed in individuals with MS [41]. Vitamin D is a potent immunomodulator [40]. Some studies have shown that administering biologically active hormone 1,25-dihydroxyvitamin D in mice inhibits the onset and progression of MS in an experimental animal model of MS [42]. The presence of VDR in neurons and microglia and CNS predicts local paracrine or autocrine effects in vitamin D [43]. A randomized double-blind, placebo-controlled trial focused on the serological markers of disease activity. Application of 1000 IE cholecalciferol for 6 months led to a significant increase in the partial reduction of anti-inflammatory cytokine transducer growth factor-β and IL-2 levels [3]. Vitamin D supplementation is recommended for patients with MS [44]. It has been reported that high-dose vitamin D supplementation has a significant effect on nerve repair, especially in demyelination caused by experimental caprizole application, and that this effect is achieved through oligodendrocyte maturation and astrocyte activation. Vitamin D supplementation is recommended for patients with MS [44]. It has been reported that high-dose vitamin D supplementation has a significant effect on nerve repair, especially in demyelination caused by experimental caprizole application, and this effect is achieved through oligodendrocyte maturation and astrocyte activation [44]. Vitamin D3 receptor has been identified in oligodendrocytes and has been suggested to be considered as a new target for the remyelination process [45]. In another study, extracellular gelsolin (GSN) and vitamin D binding protein (DBP) levels were found to be low, and it was suggested that increasing GSN levels could be a new strategic approach in treatment [46]. The most important complex conditions in MS are inflammatory demyelination and neurodegeneration. Therefore, immune system modulation and the protective effect of vitamin D are prominent factors in the treatment [47]. The most notable finding relevant to MS is that D3 promotes stem cell proliferation and drives the differentiation of neural stem cells into oligodendrocytes, which is very important for remyelination. Vitamin D intake and lower MS incidence go hand in hand [48]. There are many factors in the progression of inflammation and neurodegeneration in MS, including vitamin D deficiency, infections, and hormonal imbalance [49]. For this reason, Vitamin D deficiency has been suggested as a major risk factor for MS [50, 51]. 1,25(OH)2D3 provided significant protection by reducing inflammation, demyelination, and neuronal loss. It has been emphasized that the increase in Beclin1 expression, the increase in the Bcl-2/Bax ratio, and the decrease in LC3-accumulation, which are important factors in apoptosis, are important in this protection. For this reason, 1,25(OH)2D3 was evaluated as a promising molecule [52]. For this reason, 1,25(OH)2D3 was evaluated as a promising molecule [52]. It has been reported that obesity reduces vitamin D status; estrogen and vitamin D collaborate to promote Treg cell dominance in females [53]. Paricalcitol (Pari) is a vitamin D2 analog. It has been reported that anti-inflammatory activities in kidney and heart diseases. Suppressing NF-κB with its inhibitor, combined with Pari, could further reduce the expression of pro-inflammatory factors and associated proteins. Pari could diminish MOG-triggered EAE, as well as macrophage and T cell activation through blocking NF-κB activation, and it has therapeutic effects in experimental mouse models with MS [54]. In MS, which is characterized by multifocal demyelination, vitamin D treatment provides myelin protection by significantly increasing the levels of sulfatide, one of the most abundant myelin lipids [55]. In MS, single-nucleotide polymorphisms (SNPs) and the interaction of TNF-α and HLA DRB1*1501 constitute a major risk factor for the development of this disease [56]. Vitamin D deficiency poses a significant risk for many central nervous system diseases, including MS [21]. Perfluoroalkyl and poly-fluoroalkyl substances (PFASs) and perfluorooctanoic acid (PFOA) were determined to competitively bind to VDR, which is significant for maintaining immune, endocrine, and calcium homeostasis [57]. Therefore, it causes a negative effect against the protective activity of vitamin D. Taking vitamin D along with the diet is generally recommended for patients with MS. For this reason, it has been reported that vitamin D supplementation affects potential calcium binding by increasing remyelination and calretinin expression [58]. In another study, it was determined that Vitamin D supplementation increased the main myelin proteins such as proteolipid protein (PLP), myelin basic protein (MBP), myelin oligodendrocyte glycoprotein (MOG), and CNP [59]. It has been determined that vitamin D and vitamin D2D exert an immunomodulatory effect on the central nervous system and peripheral organs. Vitamin D serum concentration is needed to suppress the aberrant immune response in MS patients [60]. Vitamin D has been suggested as an immunomodulatory agent against the potential for multiple sclerosis in clinical disease [61]. Therefore, 300.000 IU D3 treatment monthly for 6 months not only significantly suppressed cell proliferation but also significantly increased transforming growth factor-beta and interleukin-10 levels.

In MS, it also causes dysfunction in general pathways through many environmental and genetic factors, also called N-glycation [62]. However, a study has shown that vitamin D application alone is not sufficient for treatment. Because there are also unknown factors affecting the Vitamin D receptor. It has been suggested that miRNA-125a-5p stands out as an important molecule here, and as a result, miR-125a-5p suppression significantly suppresses the decrease in vitamin D receptor in experimental autoimmune encephalomyelitis (EAE) [63].

In an experimental study, it was determined that D3 treatment stimulated neural stem cell proliferation and oligodendrocyte differentiation [64]. Only proinflammatory cells in the presence of TNF-α generated vitamin D, resulting in repression of MerTK expression and function. This selective production of calcitriol in proinflammatory myeloid cells has the potential to reduce the risk [65]. In an in vitro study, D3 up-regulated the vitamin D receptor in human astrocyte culture and also increased the vitamin D receptor in the white matter in the brain of patients with MS. It has been determined that it also stimulates [66]. Another study emphasized that vitamin D(3) plays an important role in T cell homeostasis, and correcting its deficiency may be important in the treatment of the disease [67]. Abbatemarco et al. (2019) determined that high vitamin D levels are important in preserving myelin content in progressive MS. Genetic IL-10R pathway defect could interact with a vitamin D3 insufficiency [68]. Early intervention with vitamin D3 can control the neuroinflammatory process that is the hallmark of EAE and MS immunopathogenesis [69]. MS is a neurodegenerative disease characterized by impaired oxidative balance, energy regulation, and immune response to myelin sheaths [70]. A previous study showed that vitamin E supplementation, along with vitamin D, affects the remyelination of endogenous progenitor cells in MS-like conditions and provides a therapeutic effect in this disease [71]. The binding of vitamin D to its specific nuclear receptors is a way to exert its function [72, 73]. Vitamin D3, compared with D2, changes the expression of a larger number of genes in OLs. Identification of genes affected by D3 in OLs should help to identify mechanisms mediating its action in MS [74]. It has been postulated that VitD3-TolDC-peptide(s) treatment is a potential strategy to restore tolerance in MS [75]. Vitamin D plays an important role in the central nervous system and participates in remyelination, however, a more detailed examination of this issue in vitamin D deficiency models will enable the subject to be detailed [76]. In an experimental study, it was reported that weekly 400 IU vitamin D supplementation for 3 weeks increased remyelination and prevented neuroaxonal and oxidative damage by protecting reactive oxygen species [77]. In a study examining the effect of diet on the central nervous system, it was determined that insufficient vitamin D intake in adults is a risk for MS [78]. Again, Mungan et al. [79] study also found that insufficient antioxidant intake is an important risk factor for MS. In MS, the target molecules are mainly myelin- and neuron/axon-derived proteins; in NMOSD, mainly aquaporin-4 expressed on astrocytes; and in AE, various proteins are expressed by neurons and axons [80]. This novel D2 function has encouraged researchers to develop vitamin D as a potential therapy for MS. Low serum vitamin D levels may be associated with elevated biomarkers of brain injury, including GFAP, NF-H, S100B, and UCHL1, in MS patients. Additionally, the potential neuroprotective effects of vitamin D appear to exhibit gender-specific differences [81].

## Vitamin D and Alzheimer’s Disease

Alzheimer’s disease (AD) is one of the basic causes of dementia worldwide and is a condition characterized by progressive loss of cognition and memory [82]. AD is a progressive neurodegenerative disease characterized by the presence of senile plaques formed by the collapse of amyloid β peptides in the brain [37]. Low vitamin D levels were predicted to be associated with impaired cognitive function and a high risk of AD [83]. The addition of vitamin D to the treatment of Alzheimer’s disease has been reported to increase cognitive functions [84]. Vitamin D deficiency was reported to significantly increase the risk of dementia and Alzheimer’s disease [85]. In another study, it was reported that the relationship between cognitive function and 25 (OH) D3 level was not completely clear [86, 87]. Vitamin D deficiency and chronic inflammation reduce cognitive performance and affect neurodegeneration in the aging population [88]. A strong correlation has also been determined between depression and decreased vitamin D levels in [82]. Vitamin D deficiency is associated with some diseases with aging and becomes more evident in the population as time goes by [89]. Vitamin D3 treatment causes significant positive effects on life expectancy and quality. It has been reported that the application of memantine together with vitamin D improves cognitive function in AD patients and provides a significant treatment effect in preventing the disease [90]. In another report, it was stated that stem-cell application together with D treatment provides significant protection against neurological diseases and that the neurogenesis process makes a positive contribution to this protection [20]. It has been reported that vitamin D levels are effective in neurological diseases, and therefore, low vitamin D levels have a significant role in the onset and progression of most neurodegenerative diseases, including AD [21]. Therefore, it is emphasized that it is important to keep vitamin D levels at a certain level throughout life and that vitamin D receptor agonists are important in Vitamin D-based studies. It has been reported that suppression of vitamin D receptors inevitably leads to aging and neurodegeneration and accelerates the neurodegeneration process together with Aβ toxicity [91, 92]. It has also been emphasized that since vitamin D affects calcium metabolism, it is also important for neuron structure and functions [93]. In another molecular study, it was determined that IL-34, stimulated by 1α,25(OH)2D3, constitutes an important mechanism in the protective activity of vitamin D in AD. It has been reported that long-term amine D deficiency causes neuron loss, which inevitably causes aging and neurodegeneration [26].

It has also been emphasized that Helicobacter pylori infection is an important factor in neurodegenerative diseases such as Parkinson’s and MS, including AD, by affecting vitamin D levels through hyperhomocysteinemia [24]. It has been reported that stimulating the production of PGC-1α in the hippocampus of mice in AD provides significant protection by significantly suppressing Aβ plaque formation by stimulating vitamin D receptors [94]. Another in vitro study reported that resveratrol application, together with Vitamin D, suppressed tau phosphorylation [95]. The positive effect of vitamin D application (1000 IU) on learning mechanisms has been determined [96]. Vitamin D also provides important protection against different neurodegenerative diseases by creating antioxidative, anti-inflammatory, and anti-apoptotic effects [28]. Grimm et al. [97] determined in their study that Vitamin D and its analogs suppressed Aβ production or Aβ-degradation in neuroblastoma cells or the brain of mice with vitamin D deficiency. They reported that this suppression affects Aβ-producing enzymes, BACE1 and γ-secretases. Therefore, the effect of vitamin D on AD has been demonstrated, and it has been suggested that it may be useful in preventing the disease. Similarly, it has been emphasized that the protective effect of vitamin D in AD is important not only in this disease but also in other neurodegenerative diseases [98].

Morello et al. [29] suggested that Vitamin D, as a neuroactive steroid, could be considered a preventive and therapeutic agent for several neurological diseases, including AD. However, in a study, the effect of vitamin D application on myelin oligodendrocyte glycoprotein in experimental autoimmune encephalomyelitis was examined, and it was emphasized that this effect should be taken into consideration in childhood and adolescence [99]. Vitamin D application also plays a corrective role in copper sulfate-induced memory loss by reducing hippocampal BACE1, p-tau, caspase-9, Bax, TNFα, and cortical Ach levels [100]. There are several factors in Post-traumatic stress disorder, among which vitamin D deficiency plays an important role [101]. Vitamin D deficiency reduces dopamine levels and leads to synuclein accumulation in vitamin D imbalance. In MS, deficiency in vitamins C and D also causes demyelination of neurons [31]. In recent studies, it has been reported that vitamin D deficiency is a general risk factor for MS, Parkinson’s disease, and Alzheimer’s [34]. Vitamin D presents a novel therapeutic approach for neuroinflammatory, neurodegenerative, and neuropsychiatric diseases [34].

Vitamin D corrects neurodegeneration by affecting signalling pathways by maintaining calcium balance [102]. Suppression of cognitive function occurs due to low levels of vitamin intake. For this reason, in addition to vitamin D2, it is recommended to take vitamin B and molecules such as folic acid as supplements [103]. Similarly, it is suggested that vitamin D supplementation may have a beneficial effect on the brain in individuals with suppressed cognitive function [103]. In the study conducted by Gezen-Ak and colleagues, a strong association was demonstrated between hippocampal tissue, VDR receptors, and vitamin D, suggesting that vitamin D deficiency may be correlated with Alzheimer’s disease [104].

Reduced serum vitamin D levels have been shown to exacerbate Aβ-associated neurodegeneration in cognitively intact older adults, indicating a potential contribution to the pathogenesis of Alzheimer’s disease [105].

A study found that there is a significant relationship between Alzheimer’s disease (AD) and vitamin levels. Vitamin C showed the largest difference between the AD and control groups, while vitamin D, E, folate, A, and B12 also exhibited notable differences. These findings suggest that vitamin deficiencies play a critical role in AD pathology and may represent a potential area for future therapeutic strategies [106].

A study examining the medication profiles of elderly patients with Alzheimer’s disease (AD), mild cognitive impairment (MCI), and nondemented (ND) individuals to assess the effects of polypharmacy on β-amyloid (Aβ) fibril formation found that Vitamin D significantly reduced Aβ fibril formation. This finding suggests that Vitamin D may act as a polyphenolic inhibitor of Aβ fibrillation [107].

## Vitamin D and Amyotrophic Lateral Sclerosis (ALS)

Progressive motor neuron destruction in ALS is accompanied by microglial activation, astrocytosis, lymphocyte infiltration, and dendritic cells [108]. ALS belongs to the family of neurodegenerative disorders and is classified as frontotemporal dementia, progressive muscular atrophy, primary lateral sclerosis, and pseudobulbar palsy [109]. Hypoparathyroidism, a treatable endocrinopathy, can rarely present clinically as ALS [110].

In the ALS-induced rat model, vitamin D has been reported to initiate axon regeneration by injection into the nerve cell [111]. It has been stated that the administration of vitamin D in the experimental rat model with ALS did not affect the age of onset of the disease and the formation of leg palsy [112]. In experimental studies, inflammation in traumatic brain injury was found to be reduced following D3 and has been confirmed to have a neuroprotective effect [15]. Another mechanism that explains the neuroprotective effect of vitamin D is that it reduces the level of reactive oxygen species (ROS) [113].

Supplementation of vitamin D protects neurons by preventing cytotoxicity and apoptosis. Prevention of Aβ toxicity, which was one of the basic components of AD-type pathology, by vitamin D treatment [91, 92].

It has been reported that Aβ triggers neurodegeneration by dramatically suppressing VDR expression [94]. Aβ1–42 affects most of the target genes. These affect the regulation of the amyloidogenic pathway in a complex manner, specifically, a general downregulation in NMDARs, ApoE, Trem2, and 1αOHase genes, and general upregulation of tau pathway-related genes [114]. Amyloid pathology has effects on the expression of miRNAs. miRNAs that play a part in the Aβ pathology and suggested Aβ as a counterpart of vitamin D at the crossroads of neuronal differentiation [115].

25-OH vitamin D deglycosylated vitamin D binding protein complex (VitD ~ dgVDBP) [116]. Curcumins restore Aβ phagocytosis by peripheral blood mononuclear cells in AD patients and Aβ clearance with upregulation of key genes, including MGAT3, VDR, and Toll-like receptors (TLRs) [117]. Inadequate intake of vitamin E, riboflavin, pyridoxine, folate, cobalamin, Ca, Zn, and Mg, including vitamin D, causes an increase in neurological problems in ALS [118]. Conjunctive impairments are a significant problem in conditions such as ALS. In recent years, it has been determined that vitamin D is an important modifying factor in correcting cognitive impairment. It has been observed that taking vitamin D at a level of 10 gn/ml affects cognition in ALS patients, and vitamin D insufficiency has negative effects on quality of life [119]. It is also stated that there should be a multidisciplinary approach in the treatment of ALS patients due to trabecular bone loss [120]. Circulating nutrients from the diet pose a risk for ALS, and vitamin E, along with Vitamin D, provide protection against the risk of ALS Substances such as linoleic acid are also recommended to prevent the risk of ALS [121]. Although the slowing effect of the ketogenic diet, along with vitamin supplementation, on the loss of motor neuron degeneration and the progression of the disease in ALS has been determined, it should also be noted that the selection of vitamins should be personalized [122]. Although vitamin D supplementation is recommended for patients with ALS, there are reports that this vitamin does not have a sufficient effect for treatment purposes [123]. CYP27B1 mRNA and protein expression are elevated in muscle fibres in denervating disease, especially ALS. Its upregulation is significant in the perturbation of vitamin D signalling [124]. It has been suggested that increased S-25OHD concentrations will unlikely reduce ALS incidence [125].

It has been suggested to prevent medical complications of vitamin D deficiency in ALS patients as well as in other populations of neurodegenerative patients, characterized by low mobility [126]. There are also studies stating that oral vitamin administration does not positively affect the prognosis of the disease [127]. Vitamin D was associated with baseline gross motor ALSFRS-R scores n [128].

A study evaluating the efficacy of Bu-Shen-Jian-Pi (BSJP), a traditional Chinese medicine, in the treatment of amyotrophic lateral sclerosis (ALS) found that BSJP treatment significantly improved ALS symptoms, slowed the deterioration of muscle function, reduced the rate of decline in forced vital capacity (FVC), and increased serum vitamin D3 levels without significant differences in side effects compared to the control group. The multifaceted effects of BSJP, including its potential to support liver and kidney function, may be a result of its regulation of vitamin D3 levels [129].

In advanced stages of amyotrophic lateral sclerosis (ALS), impaired respiratory function, dysphagia, and immobility can increase the risk of infections, thereby predisposing patients to sepsis as a secondary complication. Vitamin D exerts neuroprotective effects against sepsis by attenuating histone-induced pyroptosis and ferroptosis. These findings highlight the potential therapeutic role of vitamin D supplementation in mitigating sepsis-associated brain dysfunction [130].

### Limitations

This review is based on previously published experimental and clinical studies. Therefore, there are some limitations, such as methodological differences between studies, variability in sample populations, and possible data gaps. The literature search was limited to English-language articles indexed in PubMed, which means that relevant studies published in other languages or databases may have been excluded. Moreover, many of the positive findings related to vitamin D are based on animal studies, and their effects in humans remain unclear. Thus, further clinical research is needed to validate the findings presented in this review.

## Conclusion

Vitamin D cannot be considered only a vitamin; however, it plays a role in many mechanisms in brain function and diseases. Vitamin D levels are closely linked to the development of certain diseases. Although there is no consensus on this issue, it is known that vitamin D has a neuron-protective effect on the brain, so it is thought that deficiency in diseases such as PD, AD, MS, and ALS may play a significant role in aetiology.

Vitamin D deficiency has negative effects on various neurodegenerative diseases. Lower serum levels of vitamin D may contribute to Alzheimer’s disease (AD) by worsening amyloid-beta (Aβ)-associated neurodegeneration in older adults. Additionally, low vitamin D levels have been linked to a dysregulated immune system and an increased risk of developing multiple sclerosis (MS). On the other hand, calcitriol, the active form of vitamin D, has anti-inflammatory and neuroprotective effects in Parkinson’s disease (PD), which may be related to its ability to enhance the expansion of regulatory T cells (Tregs).

Research indicates that vitamin D may reduce oxidative stress in patients with multiple sclerosis, and various doses of vitamin D have shown beneficial effects on oxidative stress in relapsing-remitting MS (RRMS). Adequate levels of vitamin D may also help prevent and manage depression and cognitive dysfunction in older adults. Despite these promising findings, research is ongoing to determine the optimal dosage and long-term benefits of vitamin D supplementation for brain function. Future studies should focus on large-scale randomized controlled trials to provide precise recommendations and clarify the mechanisms behind the neuroprotective effects of vitamin D.

## Data Availability

No datasets were generated or analysed during the current study.
